# Hybrid *de novo* whole-genome assembly and annotation of the model tapeworm *Hymenolepis diminuta*

**DOI:** 10.1038/s41597-019-0311-3

**Published:** 2019-12-03

**Authors:** Robert M. Nowak, Jan P. Jastrzębski, Wiktor Kuśmirek, Rusłan Sałamatin, Małgorzata Rydzanicz, Agnieszka Sobczyk-Kopcioł, Anna Sulima-Celińska, Łukasz Paukszto, Karol G. Makowczenko, Rafał Płoski, Vasyl V. Tkach, Katarzyna Basałaj, Daniel Młocicki

**Affiliations:** 1 Institute of Computer Science, Warsaw University of Technology, Warsaw, Poland; 2 University of Warmia and Mazury, Olsztyn, Poland; 3 Department of General Biology and Parasitology, Medical University of Warsaw, Warsaw, Poland; 4 Department of Parasitology and Vector-Borne Diseases, National Institute of Public Health – National Institute of Hygiene, Warsaw, Poland; 5 Department of Medical Genetics, Medical University of Warsaw, Warsaw, Poland; 6 Department of Biology, University of North Dakota, Grand Forks, USA; 7 Witold Stefański Institute of Parasitology, Polish Academy of Sciences, Warsaw, Poland

**Keywords:** Mitochondrial genome, Eukaryote, DNA sequencing

## Abstract

Despite the use of *Hymenolepis diminuta* as a model organism in experimental parasitology, a full genome description has not yet been published. Here we present a hybrid *de novo* genome assembly based on complementary sequencing technologies and methods. The combination of Illumina paired-end, Illumina mate-pair and Oxford Nanopore Technology reads greatly improved the assembly of the *H*. *diminuta* genome. Our results indicate that the hybrid sequencing approach is the method of choice for obtaining high-quality data. The final genome assembly is 177 Mbp with contig N50 size of 75 kbp and a scaffold N50 size of 2.3 Mbp. We obtained one of the most complete cestode genome assemblies and annotated 15,169 potential protein-coding genes. The obtained data may help explain cestode gene function and better clarify the evolution of its gene families, and thus the adaptive features evolved during millennia of co-evolution with their hosts.

## Abstract (summary)

**Table:** 

Measurement(s)	whole genome sequencing assay • transcription profiling assay • sequence_assembly • sequence annotation • mitochondrial DNA
Technology Type(s)	DNA sequencing • RNA sequencing • genome assembly • bioinformatics analysis
Sample Characteristic - Organism	Hymenolepis diminuta

Machine-accessible metadata file describing the reported data: 10.6084/m9.figshare.10283018

## Background & Summary

The study of the genomics and transcriptomics of parasite model species has led to advances in the basic aspects of parasite biology, as well as new trends in human and veterinary medicine. Modern genomic tools, especially those based on a combination of multiple methods, allow detailed analyses of genome structure.

Our study used hybrid genome sequencing to examine the genome of the tapeworm *Hymenolepis diminuta* by three technologies: Illumina sequencing pair-end, Illumina mate-pair and MinION Oxford Nanopore DNA sequencing. *H*. *diminuta* is a well-described representative of the class Cestoda, the large group of parasitic flatworms that includes members known to be serious pathogens of vertebrate animals and humans^1,2^.

*H*. *diminuta* was chosen for the present study since it is commonly used in studies of new therapeutics, biochemical processes, immune responses and other host-parasite interrelationships during cestodiasis^3–8^ and is considered the most important model species in experimental cestodology. *Hymenolepis diminuta* has a worldwide distribution as an intestinal parasite of rodents (primarily rats) and humans^9^, and the tapeworms of the genus *Hymenolepis* are considered to be among the most frequent causative agents of the human cestodiasis^1^.

Despite its importance as a model organism, the genome of *H*. *diminuta* is available only as a draft genome acquired as part of the 50 Helminth Genomes project initiative^10^. In addition, Gauci *et al*.^11^ using the example of *Echinococcus granulosus*, highlight the possible limitations of published draft genomes of selected tapeworm species, one being the fact that they were sequenced using only Illumina short reads technology. Therefore, the ultimate goal of our study was to improve the accuracy of the draft genome of *H*. *diminuta*, by integrating data from three complementary approaches; this approach may significantly enhance the scientific value of the achieved datasets for future studies.

The combination of the recent progress in sequencing technologies and unlimited access to genomic data has fueled rapid development in the biomedical sciences, including parasitology. Most recently, the International Helminth Genomes Consortium released draft genomes (both published and unpublished) to present lineage-specific trends rather than individual species-specific differences^10^. This dataset of helminths genomes provides a number of new details important in studies of parasitic worms; however, there is an urgent need to continue helminth genome sequencing and improve the available genomes. An examination of the number and organization of EG95 *E*. *granulosus* vaccine-related encoding genes based on two available *E*. *granulosus* draft genomes published by Tsai *et al*.^12^ and Zheng *et al*.^13^ indicates that the genome sequence data available for *E*. *granulosus* offers limited potential for practical use^11^; in part, it was not possible to map any of the EG95 gene family members previously characterized by cloning and sequencing genomic DNA fragments. These results have revealed limitations in available genomic data and highlights deficiencies present in current genomic resources, and thus, reinforced the need to supplement available datasets with new sequencing results.

This can be achieved by simultaneous use of available sequencing technologies, providing both short and long reads. In recent years, such a hybrid approach has proven to be useful in improving quality of genome assemblies and improving discovery of gene family expansions. For instance, a hybrid approach was introduced for *de novo* human genome sequence^14^, one of the best described genomes. When assembling clownfish genome using high-coverage Illumina short reads and low-coverage Nanopore long reads, Tan *et al*.^15^ observed substantial improvement in the genome statistics when compared with Illumina-only assembly. They suggest that development and improvement of Nanopore technology will shift toward the use of high-coverage long read-only assembly, followed by multiple iterations of genome polishing using Illumina reads. Genome improvements due to the use of hybrid sequencing have been applied to characterize the genetic polymorphism in *Wuchereria bancrofti* populations, and provide, among others, a list of genetic markers useful for monitoring changes in parasite genetic diversity^16^.

The present paper provides the first results of hybrid *de novo* whole-genome sequencing of *H*. *diminuta* combined with RNAseq analysis. Our assembly appears to be more complete than that available in WormBase ParaSite^17^ and offers improved genome statistics. In this respect our results suggest that the procedure yielded one of the most comprehensive tapeworm genome assemblies available. In addition, our results are supported with RNA-seq analyses, which allow a better overview of the entire structure of the *H*. *diminuta* genome.

Here we confirm that the hybrid sequencing approach is the optimal method for obtaining the high quality data resulting in determination of a complete genome sequence. This cost-effective approach combining Illumina paired-end, mate-paired, and MinION Nanopore long reads allowed the retrieval of one of the most comprehensive tapeworm (or any parasitic worm) genome available, complimented by RNA sequencing data. These may result in better understanding of the biology of the parasite, its genetic diversity, adaptation to parasitic way of life and may allow new treatments and/or diagnostic tools to be identified in the near future.

## Methods

### Experimental animals

Approximately three month old male Lewis rats (*Rattus norvegicus domesticus*) were used as definitive hosts for adult *H*. *diminuta*. The rats were kept in plastic cages in the laboratory animal facilities of the Medical University of Warsaw, Poland. Food and water were provided *ad libitum*. This study was approved by the 3^rd^ Local Ethical Committee for Scientific Experiments on Animals in Warsaw, Poland (Permit Number 51/2012, 30^th^ of May 2012).

### Cultivation of *H*. *diminuta* adult cestodes

Six-week-old *H*. *diminuta* cysticercoids were removed from dissected *Tenebrio molitor* beetles under a microscope (100× magnification). Ten three-month-old rats were infected by voluntary oral uptake of six cysticercoids of *H*. *diminuta* per rat. Smears of their fecal samples were examined under a microscope (magnification 400×) five to six weeks from the initial infection, to verify the presence of adult parasites by their eggs. The rats were euthanized with 100 *mg*/*kg* intraperitoneal thiopental anaesthesia (Biochemie GmbH, Austria). The small intestines were removed immediately, adult parasites were isolated and washed up to 5× with 100 *mM* PBS with antibiotics added (1% penicillin) to remove debris.

### DNA isolation

Briefly after recovery from host intestine, DNA was isolated from tapeworm fragments containing only scolex and immature proglottids. Genomic DNA was isolated using a Genomic Midi AX isolation kit with ion-exchange membranes (A&A Biotechnology, Gdynia, Poland) according to the manufacturer’s instructions. The integrity of the genomic DNA molecules was checked using agarose gel electrophoresis. The obtained DNA extracts were used immediately or stored at −20 °*C* until use.

### RNA isolation and sequencing

A total of three adult *H*. *diminuta* tapeworms were homogenized in RLT buffer and total RNA was isolated from the homogenate using RNeasy Midi Kit (Qiagen, Germany). The sequencing library was prepared from 1 *μg* total RNA using TruSeq RNA Sample Preparation v2 Kit (Illumina, San Diego, CA, USA) according to manufacturer’s instructions; the library was paired-end sequenced (2 × 100 *bp*) on the Illumina HiSeq 1500 platform.

### WGS library preparation and sequencing

For whole genome sequencing (WGS) 2.5 *μg* of high quality genomic DNA was used. Prior to the library preparation DNA was fragmented using Covaris M220 (Covaris, Inc, Woburn, MA, USA) and size selection was performed using BluePippin (Sage Science, Inc, Beverly, MA, USA) for the average insert size 600 *bp*. The library was prepared using NEBNext Ultra^®^ II DNA Library Prep Kit (New England BioLabs, Inc, Ipswich, MA, USA) according to manufacturer’s instruction.

For mate-pair whole genome sequencing (MP-WGS) two different libraries, with (4 *μg* input DNA) and without (1 *μg* input DNA) size selection, were prepared. Libraries were constructed using Nextera Mate Pair Library Preparation Kit (Illumina) according to manufacturer’s instruction. Size selection was performed using BluePippin (Sage Science) for fragments ranging from 5000 to 10000 *bp* (average size 8000 *bp*). The mean fragment size for the library without size selection was 2000 *bp*.

The WGS library was paired-end sequenced on a HiSeq. 1500 (Illumina) (S59, S66, S70, S13, S41, S34, S47: 2 × 100 bp, Table 1) and on an MiSeq (Illumina) (S36: 2 × 300 bp, S3: 2 × 250 bp, Table 1). S1 was single-read sequenced (1 × 500 bp) on an MiSeq (Illumina) (Table 1). The MP-WGS library was paired-end sequenced (2 × 100 bp) on a HiSeq 1500 (Illumina).

**Table 1:** PET statistics.

Dataset	Sum	Read length	Insert size	
	[Mbp]	[bp]	Mean [bp]	Median [bp]
S59	939.647	100	338	286
S66	855.123	100	373	406
S70	6926.560	100	341	291
S13	1022.311	100	316	256
S41	14051.850	100	465	464
S34	431.827	100	337	284
S36	2438.883	300	473	467
S47	57796.150	100	463	463
S3	9029.885	250	467	463
S1	488.355	500	—	—

The set of datasets S59, S66, S70, S13, S41, S34 and S36 is PET1, whereas the set with S3 and S47 is called PET2.

For Oxford Nanopore sequencing (ONT) high molecular DNA was isolated from tapeworm using phenol-chloroform extraction. Briefly, 200 *mg* of tapeworm tissue sample was washed twice with PBS buffer to remove excess rat stool material. After washing, the sample was submerged in 900 *μl* of TE buffer. The sample was lysed by the addition of 90 *μl* of 10% SDS, 10 *μl* of Proteinase K (20 *mg*/*ml*) and incubated at 37 °*C* for one hour until all cells were lysed. Following this, 200 *μl* of 5 *M* NaCl was added to the cleared lysate, which was subjected to phenol:chloroform:isoamyl alcohol extraction until no protein debris was visible in the interphase. After protein removal, the DNA was precipitated with isopropanol (0.7 volume added) and centrifuged for 10 minutes at 14000 rpm and washed with 70% ethanol. The DNA pellet was dried for a short time at room temperature and re-suspended in 100 *μl* of low-TE buffer (10 *mM* Tris and 0.1 *mM* EDTA *pH* = 8.0) containing RNase (50 *μg*/*ml*). DNA quality and integrity were checked using electrophoresis in standard 1% agarose gel and by PFGE using Biorad CHEF-II instrument. DNA quantity was measured with Qubit 3.0 fluorimeter and Broad Range chemistry (Thermo Scientific, Life Technologies).

The Oxford nanopore library was constructed by 1D ligation using two strategies. In the first, 8 *μg* of DNA was sheared into 20 *kbp* fragments using Covaris g-Tube and 5 *μg* of sheared template was taken for 1D library construction using SQK-LSK108 kit (Oxford Nanopore Technologies). Approximately 1 *μg* of library was loaded into R9.4 flowcell system and sequenced on a MinION instrument for 24 hours. In the second approach, 20 *μg* of DNA was sheared into 20 *kbp* fragments followed by size selection on BluePippin instrument (Sage Science). Fragments above 10 *kbp* were recovered using PAC 30 kb cassette. 5 *μg* of recovered DNA was taken for 1D library construction using SQK-LSK108 kit and 1.5 *μg* of final library was loaded into R9.4.1 flowcell and sequenced on MinION sequencer.

### *De novo* genome assembly

A hybrid assembly approach was employed, with several types of reads used in the assembly. Firstly, the datasets created from high quality of DNA reads from Illumina paired-end and Illumina mate-pair sequencing were assembled using tools based on de Bruijn graph, ABySS^18^ and dnaasm^19^. The software versions are reported in Table 2. Secondly, the set of contigs (results of assembly) were combined based on the Oxford Nanopore long reads using two different tools: LINKS^20^ and dnaasm-link^21^. This step was developed in an iterative way: firstly, results obtained from only short DNA reads were linked, where distance parameter in LINKS tool was set to 6 *kbp*. The obtained results were linked with those obtained for distance values of 7 *kbp*, then 8 *kbp*, 19 *kbp*, 20 *kbp* and 30 *kbp*. However, as the LINKS application requires a very large amount of RAM, the procedure was performed using dnaasm-link running on C++ instead of Perl. In addition, dnaasm-link has a module to fill the gaps between contigs using sub-sequences from long DNA reads.

**Table 2:** Software with package version.

Name	Version	Url
BBmap^39^	38.41	https://jgi.doe.gov/data-and-tools/bbtools
FastQC^40^	0.11.8	https://www.bioinformatics.babraham.ac.uk
MultiQC^41^	1.7	https://multiqc.info/
Albacore	2.3.1	https://omictools.com/albacore-tool
NanoFilt^42^	2.2.1	https://github.com/wdecoster/nanofilt
Porechop	0.2.4	https://github.com/rrwick/Porechop
NanoPlot^42^	1.23.1	https://github.com/wdecoster/NanoPlot
Jellyfish^43^	2.2.9	https://www.cbcb.umd.edu/software/jellyfish
GenomeScope^44^	1.0.0	http://qb.cshl.edu/genomescope
BUSCO^45^	2.0	https://busco.ezlab.org
Circoletto^46^	20180728	https://github.com/infspiredBAT/Circoletto/
Trimmomatic^47^	0.38	http://www.usadellab.org/cms/?page=trimmomatic
Trinity^48^	2.8.4	https://github.com/trinityrnaseq
STAR^49^	2.4.0	https://github.com/alexdobin/STAR
BRAKER2^50^	2.1.2	https://github.com/Gaius-Augustus/BRAKER
Augustus^51^	3.2.3	http://augustus.gobics.de/
MAKER2^52^	2.31.10	https://www.yandell-lab.org/software/maker.html
Transdecoder^53^	2.0.1	https://github.com/TransDecoder/TransDecoder
g:Profiler^54^	rev 1760	https://biit.cs.ut.ee/gprofiler/gost
Trinotate^55^	3.0.2	https://github.com/Trinotate
Hmmer^56^	3.2	http://hmmer.org/
Pfam^57^	32.0	https://pfam.xfam.org/
Rnammer^58^	1.2	http://www.cbs.dtu.dk/services/RNAmmer/
SignalP^59^	4.1	http://www.cbs.dtu.dk/services/SignalP/
CLC Main Workbench	6.9.1	https://www.qiagenbioinformatics.com
MacVector	16.0.10	http://www.macvector.com/

### Functional annotation

The annotation pipeline was run using newly-obtained transcriptomic and genomic data from *H*. *diminuta*. During the first step, the RNA-seq data were mapped to the assembled genomic scaffolds using the STAR aligner. Obtained BAM file and genomic scaffolds were analyzed with BRAKER2 software with the Augustus tool to acquire the protein-expressing coding sequences. In the next step, BRAKER2 (amino acid sequences) and Trinity (transcriptomic sequences) outputs were used to obtain detailed genomic annotations using using MAKER2 pipeline (with -est2genome = 1; -prot2genome = 1).

All *de novo* assembled transcripts were searched against UniProt/SwissProt^22^ database using BLASTx and BLASTp with an *e*-*value* < 10^−5^. Open reading frames (ORFs) were predicted using Transdecoder. The remaining functional annotation was obtained using g:Profiler and Trinotate pipeline, which uses several software packages: Hmmer, a protein domain identification (Pfam) tool, Rnammer to predicts ribosomal RNA and SignalP to predicts signal peptide sites.

### Mitochondrial genome

Mitochondrial DNA was obtained and sequenced with Illumina technology as described above. The mtDNA was bioinformatically obtained from *de novo* assembly from the PET1 dataset (S59, S66, S70, S13, S41, S34 and S36 sets of reads). The mitogenome was analyzed and and characterized using CLC Main Workbench and MacVector software. The organization of mitochondrial genome is given in the ‘Technical validation’ section, where it is also compared with NC_002767.

## Data Records

Data supporting the results of this article has been deposited at European Nucleotide Archive (EMBL). The study titled ‘Hybrid sequencing of *Hymenolepis diminuta* genome’ got Access Number ERP113437^23^, the project identifier is PRJEB30942. Raw Illumina and Nanopore reads have been given the indexes ERS3052629–ERS3052634, the assembly output is deposited under name ‘H.diminuta_WMSil1’ and identifier GCA_902177915^24^, mitochondrial genome under name ‘Hymenolepis diminuta strain WMSil1 genome assembly, organelle: mitochondrion’, LR536429^25^. Annotation is included. Supporting data, also including script parameters, are available at figshare^26^.

## Technical Validation

### Paired-end reads

Firstly, the quality of input data was checked using FastQC tool. The results confirmed the high quality of DNA reads – the reports were collected by the MultiQC tool and are available online at 10.6084/m9.figshare.8798111.v1. Following this, the basic statistics of the paired-end tags were studied using the BBmap package (Table 1).

Further analysis used two data sets: PET1 and PET2. PET1 is a set with coverage 150× created from S59, S66, S70, S13, S41, S34 and S36, while PET2 was created from S3 with S47, and has 370× coverage.

### Mate-pairs reads

MP-WGS sequencing identified two sets of reads: MP1 and MP2. Both data sets consist of 100 *bp* DNA reads; however, MP1 was found to include 69, 558, 283 raw pairs of reads while MP2 had 54, 688, 723. Read quality, determined by FastQC, showed problems with adapter content, over-represented sequences and per sequence GC content, which is typical for this type of sequencing. To overcome this issue, the NxTrim^27^ tool was used to filter only correctly paired reads based on the adapter location (Table 3). The resultant sets of DNA reads were again checked by FastQC, and no such problems with DNA reads were observed.

**Table 3:** NxTrim statistics.

	MP1	MP2
Mate-pairs orientation	35.52%	35.33%
Paired-end orientation	25.22%	24.93%
Unknown orientation	38.47%	38.90%
Single end reads	0.79%	0.84%

**Fig. 1:**
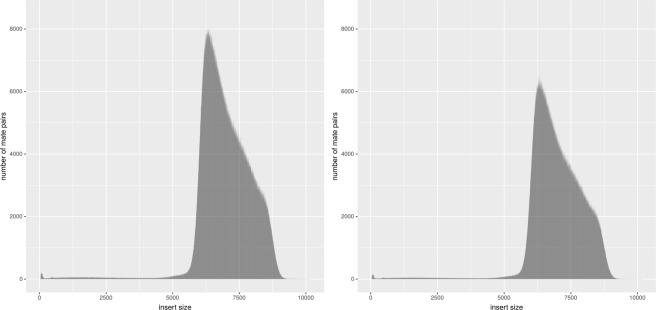
MP dataset after NxTrim trimming insert size histogram. The graphs on the left and right present the histograms for MP1 and MP2 datasets, respectively.

After rejecting improperly paired DNA reads, the insert size value of the remaining mate pairs were examined using BBmap package. The results are presented in Fig. 1.

### ONT reads

Sequencing of nanopore library without size selection (ONT1) yielded 546,222 reads and 3.5 *GB* of sequence data with a mean read length of 6.37 *kbp*. Size selected library (ONT2) sequencing yielded 156,168 reads and 1.6 *GB* of sequence data with the mean read length of 10.1 *kbp*. Nanopore sequencing yielded totally 702,390 reads and 5.1 *GB* of data.

**Fig. 2:**
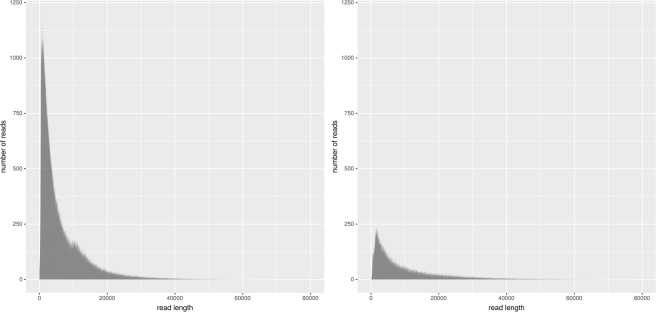
Raw ONT dataset length histogram. The graphs on the left and right present the histograms for ONT1 and ONT2 datasets, respectively.

**Fig. 3:**
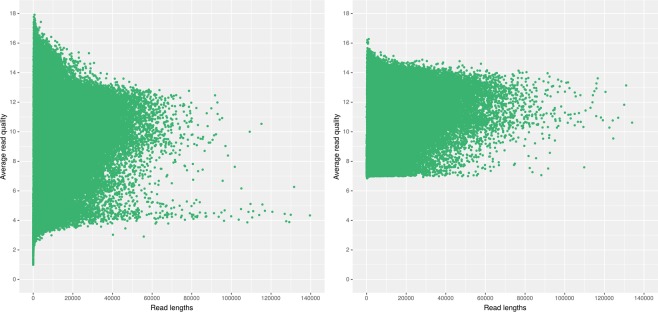
Raw ONT dataset quality diagrams. The graphs on the left and right present the diagrams for ONT1 and ONT2 datasets, respectively.

Raw nanopore data was base-called using Albacore (Oxford Nanopore Technologies, Oxford, UK). After quality filtering for quality and residual adapter removal using NanoFilt and Porechop. Long nanopore read data statistics, generated using NanoPlot, are presented in Figs. 2 and 3.

The error rate was checked using the BBmap package by mapping long DNA reads to contigs produced only from paired-end tags. The obtained results indicated a 25% error rate from nanopore DNA reads; therefore, the raw DNA reads were corrected using a Canu^28^ correcting module, resulting in the error rate falling to 11%.

### *De novo* assembly results

A hybrid assembly approach was employed, where short paired-end reads PET1 and PET2 datasets (depicted in Table 1) and mate-pair reads MP1 and MP2 (given in Table 3) and ONT1 and ONT2 long reads were used together.

The present study investigates the effect of applying reads from third-generation sequencers on *de novo* assembly results. In a typical *de novo* project, sequencing and assembly are performed iteratively until the results are of good enough quality and funds still remain. During each iteration, sufficient funds need to remain available for the next sequencing process, because the assembly costs are lower. From this point of view, two approaches can be used when performing a new experiment: (1) use the sequencing technology previously used in the project, or (2) complement results with sequencing technology not used previously in the project. Our results indicate that option (2) is a better choice, as adding results from new sequencing technology gives better statistics than additional reads obtained by the previously used technology.

As depicted in Table 4 we observed a significant improvement in assembly results between column 2 and column 3, when mate-pair reads were added, and between column 4 and column 5, when Nanopore reads were added. The improvements in assembly between column 2 and column 1, between column 4 and column 3, and between column 6 and column 5 were less pronounced since they were obtained using the same sequencing methods. In particular, using N50 statistics as a measure of quality, we observed 21% better results (from 69.7 kbp to 84.2 kbp) when using PET1 + PET2 reads instead of PET1 reads (the sequencing coverage increases from 150× into 520×). Adding the mate-pair MP1 dataset (sequencing coverage 77×) into PET1 + PET2 (dataset has 597× coverage instead of 520×) improved N50 by 1000% (from 84.2 kbp to 842.2 kbp). The next mate-pair dataset, MP2 (sequencing coverage 61×, therefore all reads cover genome 658×) improved N50 by 0.2% (from 842.2 kbp to 844.2 kbp). Using the Nanopore dataset (ONT1, coverage 19×, mean read length 6.4 kbp) improved N50 by 206% (from 844.2 kbp to 1.7 Mbp), and the next Nanopore dataset ONT2 (coverage 8×, mean read length 10.1 kbp) improved N50 by 134%. A similar effect was observed when using a number of scaffolds.

**Table 4:** The impact of sequencing strategy on *de novo* assembly results.

Assembly set:	PET1	PET1 + PET2	PET1, 2 + MP1	PET1, 2 + MP1, 2	PET1, 2 + MP1, 2 + ONT1	PET1, 2 + MP1, 2 + ONT1, 2
Number of scaffolds	4805	4688	2346	2342	902	719
Total scaffolds size [Mbp]	162.29	162.89	170.80	170.84	176.55	177.07
Longest scaffold [Mbp]	0.439	0.487	3.8	3.8	6.78	6.94
N50 scaffold [kbp]	69.7	84.2	842.2	844.2	1737	2331
Number of contigs	7424	6487	7049	7050	7127	7118
Total contigs size [Mbp]	162.12	162.78	167.66	167.66	167.93	167.95
Longest contig [kbp]	265.4	472.6	472.6	472.6	472.6	472.6
N50 contig [kbp]	46.3	56.1	73.5	73.5	75.0	75.1
Complete (BUSCOs)	630 (64.4%)	628 (64.2%)	646 (66.0%)	647 (66.2%)	649 (66.4%)	646 (66.0%)
Complete and single-copy	621 (63.5%)	620 (63.4%)	637 (65.1%)	636 (65.0%)	639 (65.3%)	638 (65.2%)
Complete and duplicated	9 (0.9%)	8 (0.8%)	9 (0.9%)	11 (1.1%)	10 (1.0%)	8 (0.8%)
Fragmented	107 (10.9%)	105 (10.7%)	92 (9.4%)	93 (9.5%)	90 (9.2%)	90 (9.2%)
Missing	241 (24.6%)	245 (25.0%)	240 (24.5%)	238 (24.3%)	239 (24.4%)	242 (24.7%)

BUSCO tool was used to compare the DNA sequence with regard to the number of reconstructed core genes. This evaluation of the DNA sequences distinguished four groups: (i) complete and single-copy, (ii) complete and duplicated, (iii) fragmented and (iv) missing core genes.

In addition, our proposed approach is cost- and time-effective, and limited basically by the access to diverse sequencing technologies.

### *De novo* transcriptome assembly

The Trimmomatic tool was used to trim out adaptors and low-quality fragments (Phred < 30) from the raw data. Reads shorter than 90 *bp* were removed from the dataset. Processed sequences were *de novo* assembled with Trinity with default parameters (k-mer = 25). This allowed to obtain a reference transcriptome comprising 28,282 transcripts. To confirm compatibility of RNA-Seq and DNA-Seq datasets, whole-transcriptiome mapping was performed to genomic scaffolds using BBMap, obtaining 85.65% (24,223/28,282) uniquely aligned transcripts.

We used the BUSCO tool on the transciptome, yielding 784 complete, 668 complete and single-copy, 116 complete and duplicated, 40 fragmented and 154 missing BUSCOs. This result is better than the results of the scaffold analysis (Table 4).

### Genome characteristics

**Fig. 4:**
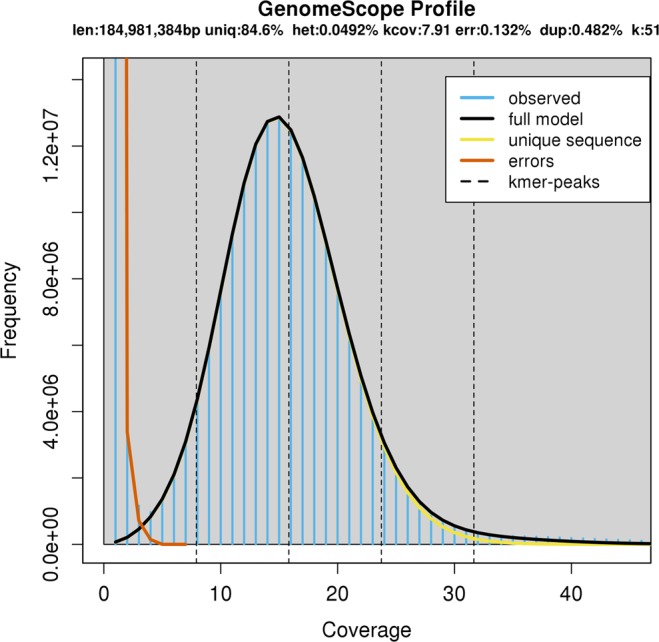
Results obtained by GenomeScope application. Shortcuts on the diagram: len – inferred total genome length, uniq – percent of the genome that is unique (not repetitive), het – overall rate of heterozygosity, kcov – mean k-mer coverage for heterozygous bases, err – error rate of the reads, dup – average rate of read duplications, k – k-mer size, observed – the observed k-mer profile, full model – estimated GenomeScope model, unique sequence – line representing unique sequences (k-mers below the line are treated as unique), errors – line representing sequencing errors (k-mers below the line are treated as incorrect), k-mer peaks – increased number of k-mers compared to the number of k-mers with lower and higher coverage.

Firstly, the k-mers distribution of the genome was studied using Jellyfish and GenomeScope tools. Jellyfish was used to obtain 51-mer count histogram in a subset of 7 *GB* of the short DNA reads, which was used to estimate genome size, heterozygosity and repeat content with the aid of GenomeScope. The size of the test genome was found to be approximately 185 *Mbp* (value close to the 177 *Mbp* resultant assembling size, see Table 5) with low heterozygosity (below 0.05%) and 15.4% repeat content (Fig. 4).

**Table 5:** A comparison of hybrid assembly results with data available at WormBase ParaSite.

Genome assembly	Our	WormBase	WormBase (≥1000 bp)
Number of scaffolds	719	13910	9867
Total scaffolds size [Mbp]	177.074	165.879	163.033
Longest scaffold [kbp]	6937	356	356
N50 scaffold [kbp]	2331	49.9	51.2
Number of contigs	7118	18736	14152
Total contigs size [Mbp]	167.947	164.748	162.069
Longest contig [kbp]	472.6	338.0	338.0
N50 contig [kbp]	75.1	38.1	38.9
Complete BUSCOs	646 (66.0%)	610 (62.4%)	611 (62.5%)
Complete and single-copy BUSCOs	638 (65.2%)	605 (61.9%)	606 (62.0%)
Complete and duplicated	8 (0.8%)	5 (0.5%)	5 (0.5%)
Fragmented	90 (9.2%)	110 (11.2%)	109 (11.1%)
Missing	242 (24.7%)	258 (26.4%)	258 (26.4%)

We tried to confirm the high repeat content value by launching the RepeatMasker^29,30^ tool with Repbase^31^ database (databases Dfam_Consensus-20170127 and RepBase-20181026). Several families of repeat elements covering only 0.72% of the genome were identified. However, in the presented genome assembly 9.127 *Mbp* of the 177.074 *Mbp* (5.2%) is known as ‘N’ signs. In addition, we estimate that approximately 8 *Mbp* (4.5%) of the genome has not been assembled. Most of the ‘N’ signs and unassembled sequences may consist of repetitive sequences, which may be a response to the high value of the predicted repeat content.

### Genome assembly results comparison to results available at WormBase ParaSite

**Fig. 5:**
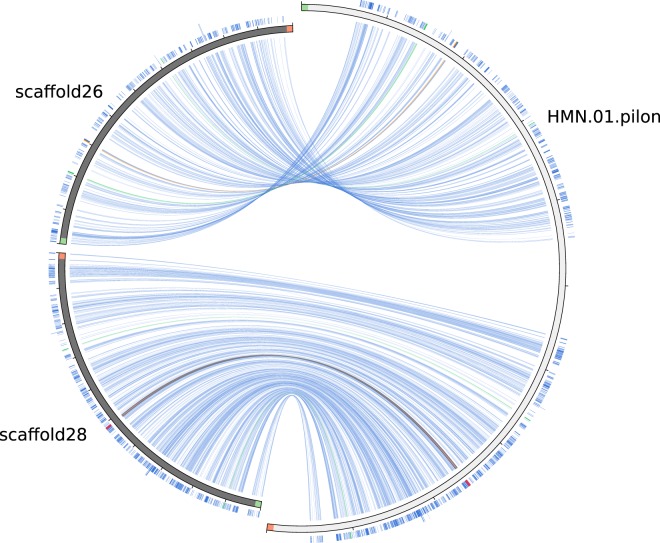
Results obtained by the Circoletto application. The presented diagram compares the HMN_01_pilon sequence (subsequence from 18 Mbp to 24 Mbp indices) from the *Hymenolepis microstoma* genome (from WormBase ParaSite) to two scaffolds from the presented study: scaffold26 and scaffold28. Colors mean identity level: blue ≤ 0.25, green ≤ 0.50, orange ≤ 0.75, red > 0.75.

The *H*. *diminuta* genome has previously been studied and the genome draft is available^10^. However, our sequencing effort^23^ resulted in approximately 45× better N50 statistics (2.3 Mbp versus 51.0 kbp; in presented study 13× fewer scaffolds were obtained: 719 scaffolds in comparison with 9867, with the longest being almost 7 *Mbp* compared to 356 *kbp* in the previous work (Table 5). Our results were also evaluated using the Circoletto tool; example results are presented in Fig. 5.

### Mitochondrial genome characteristics

**Fig. 6:**
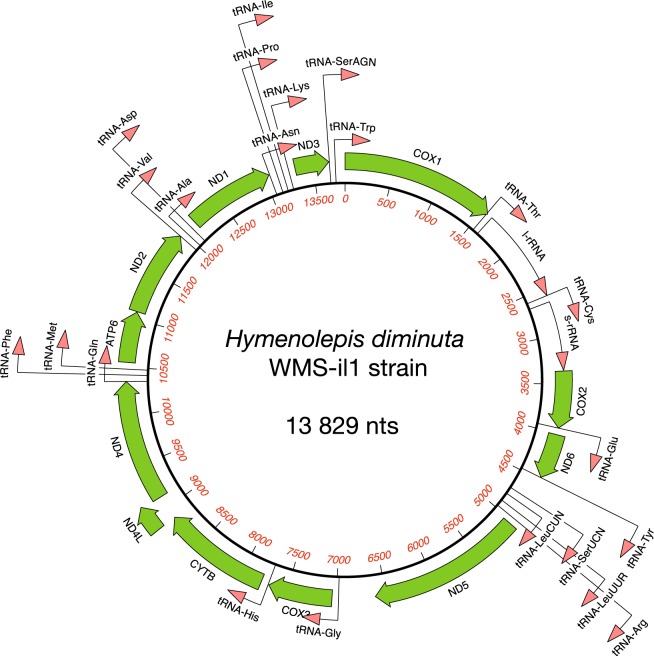
The organization of mitochondrial genome of *Hymenolepis diminuta* (WMS-il1 strain). All genes are transcribed in the same direction. The two leucine tRNA genes are designated by tRNA-LeuCUN and tRNA-LeuUUR, respectively, and two serine tRNA genes by tRNA-SerUCN and tRNA-SerAGN, respectively. Gene scaling is only approximate.

Our results indicate that the complete mitogenome of *H*. *diminuta* WMS-il1 strain consists of 13,829 *bp*, and includes 36 genes: two rRNA genes (l-rRNA, s-rRNA), 22 tRNA genes (Ala, Arg, Asn, Asp, Cys, Gln, Glu, Gly, His, Ile, Leu-1, Leu-2, Met, Lys, Phe, Pro, Ser-1, Ser-2, Thr, Trp, Tyr, Val), and 12 protein-coding genes (*atp6*, *cox1*, *cox2*, *cox3*, *cytb*, *nad1*, *nad2*, *nad3*, *nad4*, *nad4L*, *nad5*, *nad6*). All identified genes are oriented in the same direction (Fig. 6).

The rrnL gene (967 *bp*) is separated from the rrnS gene (709 *bp*) by the tRNA-Cys gene. The length of the tRNA genes vary from 59 *bp* (tRNA-Ser) to 72 *bp* (tRNA-His). The 12 protein-coding genes encoded a total number of 3,363 amino acids. The total length of all protein-coding genes was found to be 10,089 *bp*. The length of the individual protein-coding genes varied from 261 *bp* (*nad4L* gene) to 1599 *bp* (*cox1* gene). Except for the *nad4* gene, all the protein-coding genes use the ATG start codon, whereas the *nad4* gene uses ATT as a start codon. The majority of identified protein-coding genes are terminated with the TAG termination codon; the only exceptions are *cox2* and *nad6* genes, which are terminated with the TAA codon.

In the mitogenome of *H*. *diminuta* (WMS-il1 strain) two non-coding regions were found: the larger between ND5 and tRNA-Gly genes, and the shorter is between the tRNA-Tyr and tRNA-Ser genes. The nucleotide composition of the obtained mitogenome is *A* = 25.4%, *T* = 45.6%, *G* = 19.3% and *C* = 9.6%.

Our mitogenome analysis of the *H*. *diminuta* WMS-il1 strain mitogenome was performed using data from the Illumina next-generation sequencing. All 36 genes previously found in mitogenomes of other cestode species were identified^32–34^. The length, structure and composition of the coding regions are also similar to these previously described in tapeworms, including NCBI *H*. *diminuta* reference sequence NC_002767.1^35^. No differences were observed in the gene sequence encoding tRNA. However, both rRNA-coding genes differed with regard to two bases when compared to the reference sequence. Interestingly, the protein-coding regions showed substantial variability and only ND3 was identical as these described in reference sequence. These differences are shown in Table 6.

**Table 6:** Organization of *Hymenolepis diminuta* (WMS-il1 strain) mitochondrial genome, and a comparison with the NC_002767 genome.

Gene/region	Position	Size [bp]	Codon		Difference
			Start	Stop	
cox1	13827–13829	1599	ATG	TAG	20 –/T, 192 T/C, 371 A/G
	1–1596				951 W/A, 1596 G/A
tRNA-Thr	1587–1651	65			
rrnL	1652–2618	967			1743 T/C
tRNA-Cys	2619–2685	67			
rrnS	2686–3394	709			2862 A/T
cox2	3401–3979	579	ATG	TAA	3462 A/G, 3886 A/G
tRNA-Glu	3980–4044	65			
nad6	4048–4506	459	ATG	TAA	4059 C/T, 4224 C/T, 4308 C/T
tRNA-Tyr	4510–4575	66			
Non-coding	4576–4758	183			
tRNA-SerUCN	4759–4825	67			
tRNA-LeuCUN	4838–4905	68			
tRNA-LeuUUR	4931–4993	63			
tRNA-Arg	5004–5063	60			
nad5	5067–6641	1575	ATG	TAG	5153 G/A, 5260 G/A
					5302 C/T, 6570 A/G
Non-coding	6642–7013	372			
tRNA-Gly	7014–7076	63			
cox3	7080–7730	651	ATG	TAG	7422 G/A
tRNA-His	7739–7810	72			
cytb	7814–8911	1098	ATG	TAG	
nad4L	8915–9175	261	ATG	TAG	9044 T/C, 9053 C/T
nad4	9160–10389	1230	ATT	TAG	9176 C/T, 9783 T/C, 10038 G/A
tRNA-Gln	10391–10456	66			
tRNA-Phe	10456–10518	63			
tRNA-Met	10515–10578	64			
atp6	10583–11098	516	ATG	TAG	10703 G/T, 10959 T/C
nad2	11105–11986	882	ATG	TAG	11482 A/G
tRNA-Val	11987–12051	65			
tRNA-Ala	12053–12122	70			
tRNA-Asp	12127–12188	62			
nad1	12189–13079	891	ATG	TAG	12330 C/T
tRNA-Asn	13088–13151	64			
tRNA-Pro	13160–13222	63			
tRNA-Ile	13222–13283	62			
tRNA-Lys	13285–13348	64			
nad3	13352–13699	348	ATG	TAG	
tRNA-SerAGN	13705–13763	59			
tRNA-Trp	13764–13829	66			

### Gene prediction

Gene prediction was performed with genomic scaffolds according to the protein sequences of *H*. *diminuta* (PRJEB507) and other closely-related organisms: *H*. *nana* (PRJEB508), *H*. *microstoma* (PRJEB124) and *Echinococcus multilocularis* (PRJEB122), downloaded from WormBase ParaSite database^36^ Version: WBPS12 (WS267). This step was processed again by MAKER2 software (with -est2genome = 0; -prot2genome = 0). The annotation files (GFF3) obtained from each species were combined and both results were compared using custom script in the R environment ver. 3.5.0. Next, CDS annotations not confirmed in either pathway which were shorter than 150 *nt* (as suggested by NCBI) were removed from the final GFF3 file using Genome Annotation Generator (GAG)^37^ with -rcs 150 option. The general statistics of GFF file modifications using GAG are presented in Table 7.

**Table 7:** Overall statistics of the genome annotation.

	Number	Total length [bp]	Longest [bp]	Mean length [bp]
gene	15169	64715316	111112	4266
mRNA	19651	97445324	111112	4959
exon	106310	24358392	18969	229
intron	86659	73086932	19840	843
CDS	19651	20703936	21660	1054
Total sequence length			177074070	
% of genome covered by genes			36.5	
% of genome covered by CDS			11.7	
mean mRNAs per gene			1.3	
mean exons per mRNA			5.4	
mean introns per mRNA			4.4	

**Fig. 7:**
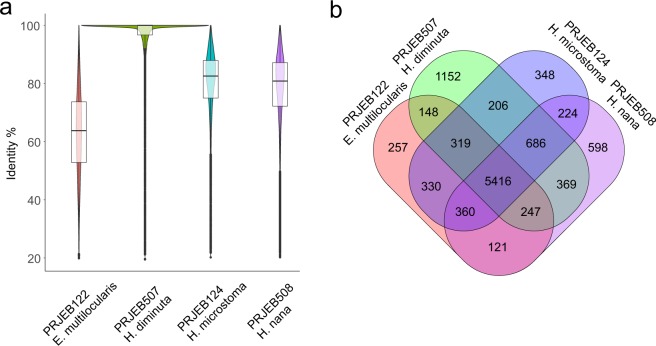
The results of bidirectional BLAST of predicted protein coding genes (proteins) against four reference proteomes. (**a**) The distribution of the *de novo* assembled protein coding sequences across four closely related cestode species. (**b**) The Venn diagram of 15,169 predicted proteins. The four included cestode species shared a core set of 5,416 proteins, a total of 8,543 proteins were included with reference to the *H*. *diminuta* proteome and 1,152 were unique for this tapeworm across all analyzed species.

A total of 15,169 potential protein-coding genes were predicted in the assembled *H*. *diminuta* genome and functionally annotated, which encodes 19,651 mRNAs. For extracting CDS sequences, the gffread (https://github.com/gpertea/gffread) script was applied. In total 16,983 (86.42%) homologs were identified in *H*. *diminuta* with a median sequence identity of 98.91%, 15,144 (77.06%) homologs in *H*. *microstoma* with a median sequence identity of 80.36%, 14,668 (74.74%) homologs in *H*. *nana* with a median sequence identity of 78.04%, and 14,132 (71.91%) homologs in *E*. *multilocularis* with a median sequence identity of 60.00% (Fig. 7(a,b)) by searching WormBase ParaSite database using BLASTp^38^ and CDS sequences as query.

### Annotation results

**Fig. 8:**
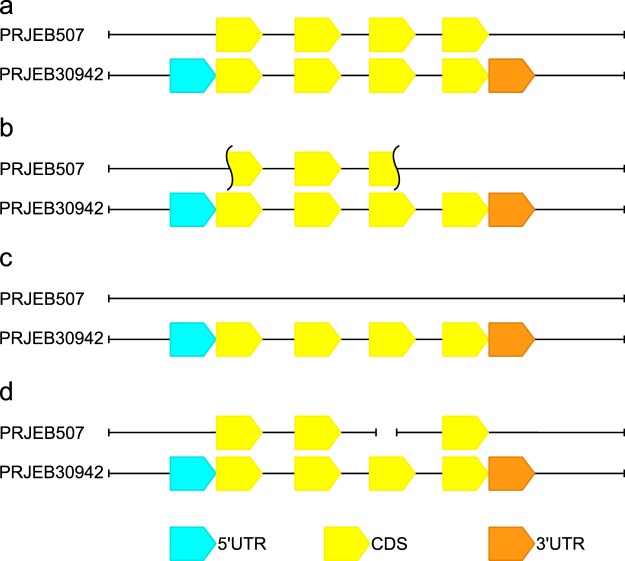
The schematic diagram showing the types of improvements in the annotation of the *H*. *diminuta* genome. (**a**) Additions to the UTR annotations; (**b**) improvement of the CDS regions; (**c**) new gene annotations; (**d**) merging of two reference annotations. More detailed diagram, including examples of improvements, is presented in the Supplementary Figure (A–D).

Our sequencing and annotation results enriched *de novo* assembly reference of the *H*.*diminuta* genome available from WormBase ParaSite. A considerable body of the annotation created only by *in silico* prediction is incomplete, and requires re-annotation. Our acquisition of RNA-seq data offers a significant improvement in the finalization of the annotation processes, as even mRNA sequences from related organisms (*H*. *microstoma*, *H*. *nana*, *E*. *multilocularis*) do not always form the best basis for exon–intron structure prediction. By using transcriptome evidence from the same species (*H*. *diminuta*) it was possible to confirm intronic donor-acceptor sites according to the alignment of cDNA and genomic DNA. Our improved annotation allowed the splice site to be corrected according to *de novo* assembly transcriptome aligned to *H*. *diminuta* genome. Software applied in this study allowed us to add UTR regions to previously-annotated genes (Fig. 8a and Suppl. A). Our data includes some fixes of the reference CDS regions (Fig. 8b and Suppl. B); in addition, the *H*. *diminuta* genome was supplemented with genes that have not yet been annotated in the reference genome (Fig. 8c and Suppl. C). In some cases, two gene annotations, predicted by Sanger Institute (annotated on two separated scaffolds, blue -HDID_scaffold0000291 and orange -HDID_scaffold0000029 bars on the Fig. 8d and Suppl. D) were merged into individual complete protein-coding gene.

## Supplementary information

annotation_supplementary

## Data Availability

The software packages used for the analysis with version numbers, are given in Table 2. The parameters of scripts used for the analysis are available at figshare^26^.
