# Author Correction: Hybrid *de novo* whole-genome assembly and annotation of the model tapeworm *Hymenolepis diminuta*

**DOI:** 10.1038/s41597-020-0394-x

**Published:** 2020-02-10

**Authors:** Robert M. Nowak, Jan P. Jastrzębski, Wiktor Kuśmirek, Rusłan Sałamatin, Małgorzata Rydzanicz, Agnieszka Sobczyk-Kopcioł, Anna Sulima-Celińska, Łukasz Paukszto, Karol G. Makowczenko, Rafał Płoski, Vasyl V. Tkach, Katarzyna Basałaj, Daniel Młocicki

**Affiliations:** 1 Institute of Computer Science, Warsaw University of Technology, Warsaw, Poland; 2 University of Warmia and Mazury, Olsztyn, Poland; 3 Department of General Biology and Parasitology, Medical University of Warsaw, Warsaw, Poland; 4 Department of Parasitology and Vector-Borne Diseases, National Institute of Public Health – National Institute of Hygiene, Warsaw, Poland; 5 Department of Medical Genetics, Medical University of Warsaw, Warsaw, Poland; 6 Department of Biology, University of North Dakota, Grand Forks, USA; 7 Witold Stefański Institute of Parasitology, Polish Academy of Sciences, Warsaw, Poland

Correction to: *Scientific Data* 10.1038/s41597-019-0311-3, published online 03 December 2019

Following publication, it was found that a part of the Acknowledgements section of this Data Descriptor was missing. This has now been corrected in the HTML and PDF versions.

