# Supplementary figures and images for: Hybrid de novo whole-genome assembly and annotation of the model tapeworm Hymenolepis diminuta
Source: Sci Data. 2019 Dec 3;6:302. doi: 10.1038/s41597-019-0311-3 (PMC6890685; doi:10.1038/s41597-019-0311-3)

a

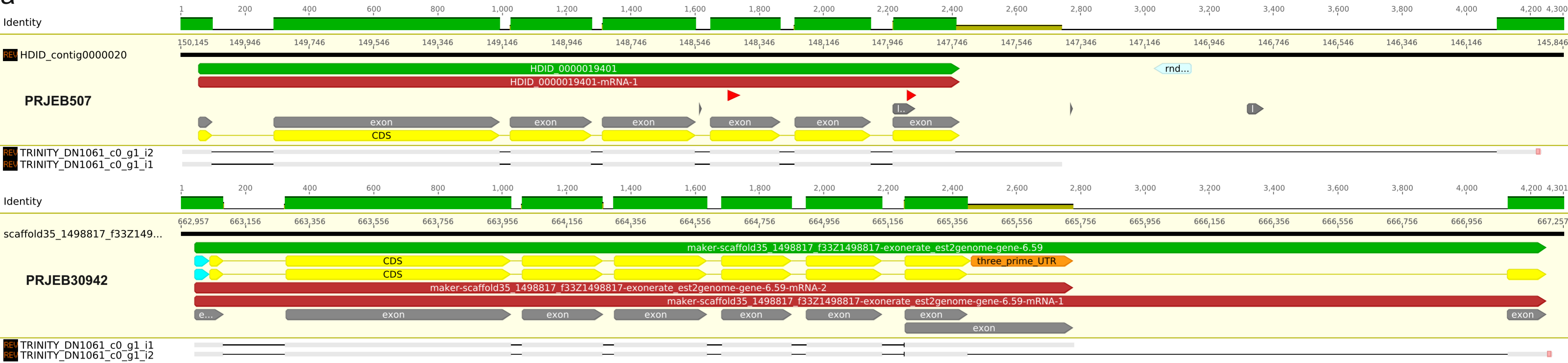

b

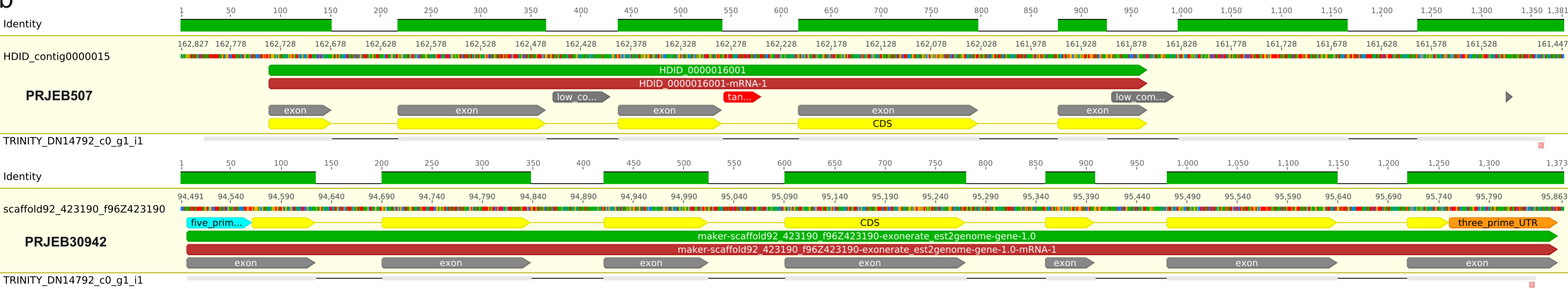

c

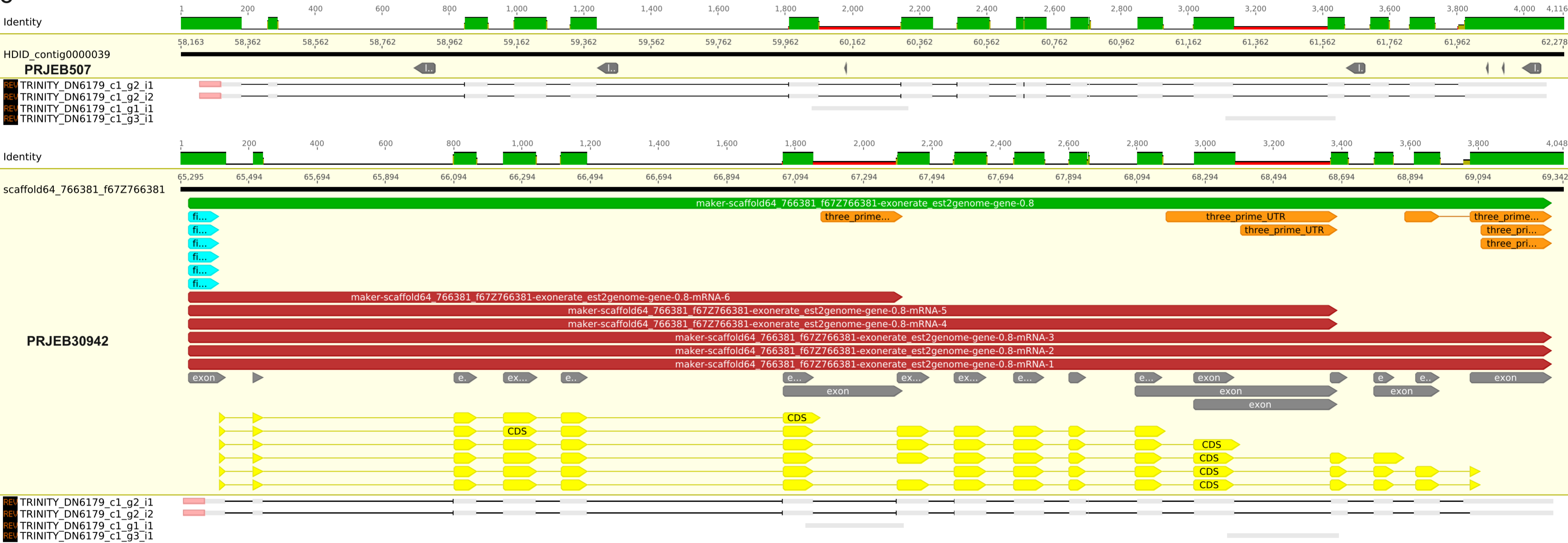

d

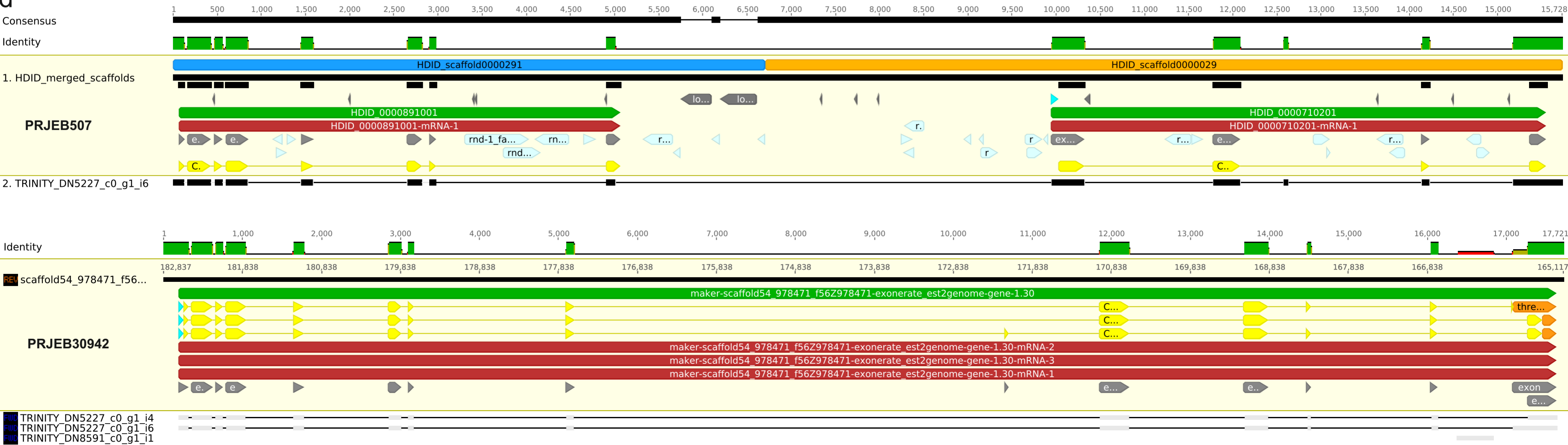

Supplement: Supplementary file 1 — annotation_supplementary [file 41597_2019_311_MOESM1_ESM.pdf]
